# Mass Spectrometry-Based *N*-Glycomics of Colorectal Cancer

**DOI:** 10.3390/ijms161226165

**Published:** 2015-12-09

**Authors:** Manveen K. Sethi, Susan Fanayan

**Affiliations:** 1 Department of Chemistry and Biomolecular Sciences, Macquarie University, North Ryde, NSW 2109, Australia; manveen.sethi@students.mq.edu.au; 2 Department of Biomedical Sciences, Macquarie University, North Ryde, NSW 2109, Australia

**Keywords:** cancer, colorectal cancer, glycosylation, glycome, *N*-glycomics

## Abstract

Colorectal cancer (CRC) is one of the most prevalent cancers worldwide. An increased molecular understanding of the CRC pathology is warranted to gain insights into the underlying molecular and cellular mechanisms of the disease. Altered protein glycosylation patterns are associated with most diseases including malignant transformation. Recent advances in mass spectrometry and bioinformatics have accelerated glycomics research and present a new paradigm for cancer biomarker discovery. Mass spectrometry (MS)-based glycoproteomics and glycomics, therefore, hold considerable promise to improve the discovery of novel biomarkers with utility in disease diagnosis and therapy. This review focuses on the emerging field of glycomics to present a comprehensive review of advances in technologies and their application in studies aimed at discovering novel glycan-based biomarkers. We will also discuss some of the challenges associated with using glycans as biomarkers.

## 1. Introduction

Colorectal cancer (CRC) is one of the most prevalent cancers, with a major worldwide burden. It usually begins as a benign lesion or polyp, which can progress slowly to cancer. CRC is a potentially curable disease if diagnosed in the early stages. If detected at an early stage, when the tumor is still localized, the 5-year survival rate is >90% following surgical resection [1]. Unfortunately, nearly half of CRC patients already have metastatic disease at presentation, when prognosis is poor with five-year survival <10%. Current CRC screening options include barium enema, colonoscopy, sigmoidoscopy and fecal occult blood testing (FOBT) [2,3]. Further, screening compliance remains low due to invasive, unpleasant nature and cost (colonoscopy) or lack of specificity and sensitivity (FOBT) [4]. There is growing expectation that new generation of screening tests based on molecular biomarkers originating from biological samples (e.g., patient blood or tissue) would provide a more sensitive, specific and less invasive alternative which would improve patient compliance [5].

Proteins are often modified by the attachment of glycans during protein synthesis. It is estimated that over 70% of all human proteins are glycosylated [6] making glycosylation the most common post-translational modification (PTM) of proteins. Glycoproteins are most commonly found in intracellular organelles (e.g., endoplasmic reticulum (ER)/Golgi), on cell surfaces and in the extracellular environment. This makes glycoproteins the primary molecular contact point for host cell–cell interactions and in pathogen invasion in the extracellular environment [7]. In mammals, carbohydrate moieties are most commonly either attached to: (i) amide nitrogen atoms of asparagine residues localized in a conserved consensus sequence (sequon) of Asn-X-Ser/Thr (where X ≠ Pro) known as *N*-linked glycosylation; or (ii) linked to the oxygen atom in hydroxyl groups of consensus-free serine or threonine residues referred as *O*-linked glycosylation [8].

Aberrant protein glycosylation is a well-known event in various disease states including cancer. Altered protein glycosylation at the cell surface or in the secretome is recognized as a factor that can cause, contribute to, or result from the development of several diseases such as congenital disorder of glycosylation, immunodeficiency and cancer [7,9,10]. In cancer, altered glycosylation is recognized as a hallmark event, with tumor-specific glycoproteins playing a pivotal role in tumor growth, migration, invasion, and metastasis [11,12,13]. Tumor-associated glycans have been studied extensively as specific tumor markers and potential therapeutic targets [11].

Driven by recent technological advances, mass spectrometry (MS)-based glycomics and glycoproteomics are gaining momentum in cancer research and hold considerable promise to uncover the biomolecular deregulations associated with cancer and identify potential markers. This review will focus on the emerging field of glycomics and present a comprehensive overview of technological advances in mass-spectrometry-based *N*-glycomics in cancer, specifically CRC.

## 2. Colorectal Cancer (CRC)

CRC arises from genetic mutations and molecular abnormalities that occur in a reasonably well-understood sequence of events [14,15]. Mutations in the adenomatous polyposis coli (*APC*) gene are an early event in CRC, believed to be associated with approximately 80% of CRC cases [16]. Other mutations associated with CRC include the microsatellite instability (MSI) condition in which the DNA mismatch repair (MMR) genes are inactivated [17], mutations in transforming growth factor β receptor II (*TGFβRII*) [16], *B-Raf* proto-oncogene [18], and beta-catenin (*CTNNB1*) [19].

CRC is the second and third most prevalent cancer in females and males, respectively, in developed countries. Globally, around 1.2 million cases and 600,000 deaths of CRC were reported in 2008 [20]. The symptoms associated with CRC, including rectal bleeding, abdominal pain and changes in bowel habits (e.g., diarrhoea or constipation), loss of weight and anaemia, are common to most CRC patients irrespective of age and gender [21,22,23], but generally lack clinical utility for early detection of CRC. Accurate and rapid diagnostic methods are therefore required to enable early CRC detection, which is critical to reduce the mortality.

Biomarkers are used for diagnosis, prognosis, and prediction of response to therapy or disease recurrence. It is generally agreed that diagnostic and prognostic markers may reduce patient mortality by yielding an accurate diagnosis and prognosis of early stage disease whereas predictive markers help to assess the patient response to a particular treatment. Potential sources of biomarkers include blood, tissues, urine and faeces. Blood and tissues remain the most widely used biological specimen for biomarker discovery studies.

Current treatment options available for CRC include surgery followed by chemotherapy, radiation therapy or a combination of both. Currently, the most widely used chemotherapeutic agent for the treatment of CRC is 5-Fluorouracil (5-FU). Over the last decade significant progress has been made in the development of more efficacious agents such as oxaliplatin, luecovorin, bevacizumab and irinotecan. 5-FU in combination with luecovorin, irinotecan or oxaliplatin [24,25,26], with or without the administration of bevacizumab [27], are some of the standard first-line treatment options available for metastatic CRC patients. Multi-agent combination therapy for treatment of CRC has been shown to improve response rate with greater progression-free survival, but associated with higher cytotoxicity than single agent administration [28].

## 3. Overview of *N*-Glycosylation and Its Biological Roles

*N*-linked glycosylation is the addition, removal and modification of monosaccharides on glycoproteins, catalyzed by various glycosyltransferases, glycosidases and other assisting glyco-enzymes. Protein *N*-glycosylation has been shown to play crucial roles in various biological processes including cell adhesion, proliferation, cellular signaling and immune response [29]. A hallmark of disease states such as cancer, aberrant glycosylation is usually caused by enzymatic perturbations in the glycosylation machinery of the affected cells [30]. Mapping the altered glycosylation machinery may improve our mechanistic understanding of the glycosylation changes and their functional relevance associated with cancer.

**Figure 1:**
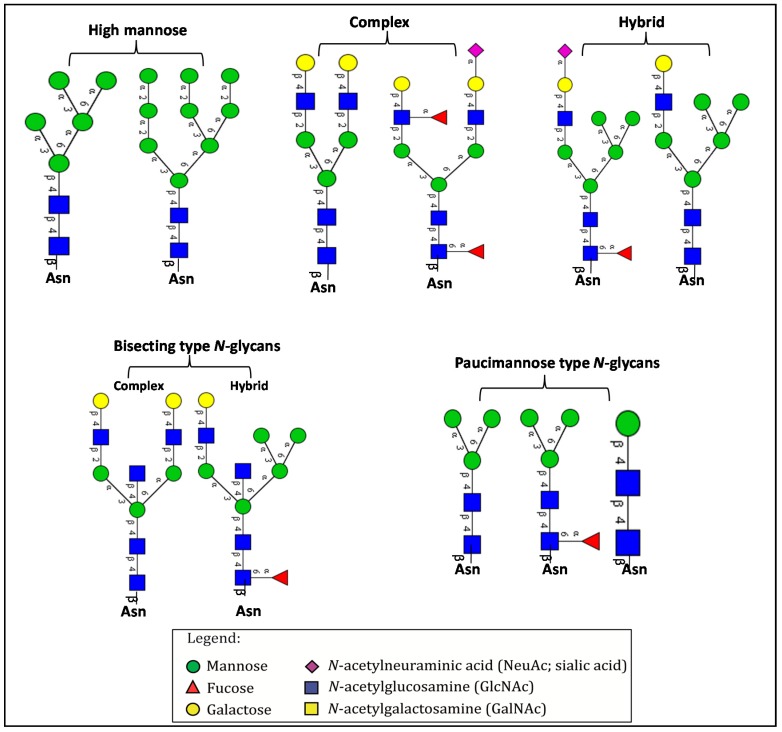
Types of *N*-glycans. Three main *N*-glycan structures in a mature glycoprotein include high mannose, complex and hybrid type. All classes share a common core, which may receive α1,6-fucosylation, bisecting β1,4-GlcNAcylation or other glycol determinants of the complex and hybrid type. Paucimannosidic type structures belong to an unusual (fourth) type of *N*-glycans in humans that may be truncated from the *N-*glycan core.

Among the known *N*-glycan linkages attached to the polypeptide backbone, the β-linked *N*-acetylglucosamine (GlcNAc) is the most prevalent type [31]. *N*-glycans of this family share a common trimannosyl chitobiose core sequence (Manα1,6(Manα1,3)Manβ1,4GlcNAcβ1,4GlcNAcβ1-Asn), which is further classified into three main classes: (i) High mannose type *N*-glycans in which only mannose residues are attached to the core; (ii) complex type *N*-glycans in which the core is extended by GlcNAc residues on both mannose arms; and (iii) hybrid type *N*-glycans in which the Manα-1,6 arm of core contains only mannose residues while the Manα-1,3 arm is extended by a complex-like GlcNAc residue, Figure 1. In addition, they also include some complex and hybrid type glycan determinants such as bisecting GlcNAc, where a β1,4-GlcNAc residue is attached to the trimannosyl core, as well as galactosylation, sialylation and fucosylation of the outer antennas. The paucimannosidic glycans represent the fourth and unusual type of *N*-glycans, which are common in invertebrates [32], but only recently reported in mammalian specimen [33,34,35,36,37].

Glycans are involved in a variety of biological roles that can be broadly divided into two categories: (i) modulatory functions of the glycans on their protein carriers; and (ii) protein carrier-independent functions of the glycans, which often involve specific recognition of glycans by glycan-binding proteins or lectins. For the latter category, it is known that glycan-binding proteins can be either of host origin e.g., involved in intrinsic cell–cell interaction, communication and recognition or alternatively of foreign origin e.g., involved in microbial adhesion and agglutination [38,39]. It is therefore well-accepted that glycans play important roles in cell–cell adhesion, cell–cell migration and cell–matrix interaction. For example, selectins mediate adhesion and interaction between blood components and vascular cells by recognizing specific glycan determinants for the endothelium attachment [40]. *N*-glycans are also involved in protein folding and quality control in the ER, ensuring proper folding of newly synthesized proteins [41].

Studies have shown that a given glycan moiety presented on different glycoconjugates may play different roles when expressed in different tissues (e.g., different stages of development). For example, mannose-6-phosphate (Man-6-P) was originally found on lysosomal enzymes and associated with lysosomal trafficking. However, Man-6-P was later identified on a variety of non-lysosomal proteins e.g., thyroglobulin, epidermal growth factor receptor (EGFR) and TGF-β precursor, which display different biological functions [42,43].

Over the past decades, numerous studies have explored the multiple and diverse functional roles of protein *N*-glycosylation [44,45]. In addition, the functional roles of *N*-glycans have been investigated by studying the naturally occurring defects in the *N*-glycosylation machinery such as congenital disorders of glycosylation. Although we now know more about the functional role(s) of protein *N*-glycosylation, this field of research remains largely unexplored, in particular in the context of the role of glycans on specific proteins.

## 4. Aberrant *N-*Glycosylation in CRC and Other Cancers

Protein glycosylation is controlled by several factors such as the nature of the polypeptide chain and the speed of its translation and folding, availability and localization of nucleotide sugar donors and substrates, competition between and differential expression of glycosyltransferase and glycosidase enzymes, and the general trafficking route and speed through the ER-Golgi network. Alterations in any of these variables may lead to cancer-associated aberrant glycosylation [46]. Some important alterations in protein glycosylation associated with cancer, including CRC, are briefly discussed below.

**Differential Expression of Glycosyltransferases:** Differential expression of glycosyltransferases and their target proteins within the tumor cell is one of the primary causes of aberrant glycosylation in cancer [9,47]. Aberrant expression of several glycosyltransferases, during carcinogenesis, has been described by several studies. Among the best characterized glycosyltransferasesis is *N*-acetylglucosaminyltransferase V (GnT-V) that catalyzes the formation of β1,6-GlcNAc (*N*-acetylglucosamine) branches on *N*-glycans. Increased expression of GnT-V and its product, β1,6-GlcNAc branched *N*-glycans are commonly observed in malignancies and considered to be positively associated with tumor growth and metastasis [48,49]. Several studies reported increased expression of β1,6-GlcNAc branched *N*-glycans in CRC and their association with increased metastasis and invasion, and decreased overall patient survival [50,51,52].

Sialyltransferases represent another important group of glycosyltransferase, which are at least partially responsible for the generation of polylactosamine residues, polysialic acid, terminal and truncated sialylated structures and some ganglioside epitopes, glycosylation features which are all linked to cancer [53]. Altered expression in cancer and contribution to tumor progression by most sialyltransferases and their associated products in cancer have been widely reported [54]. Elevated levels of total sialylation, in particular α2,6-sialylation, has been observed in several CRC studies [33,35,55,56,57]. Interestingly, increased expression of *ST6GAL1* (responsible for α2,6-sialylation) has been associated with CRC progression, invasion and metastasis [58,59], supporting the functional involvement of sialylated glycol determinants in malignant transformation processes. In addition, increased expression of certain sialylated glycan epitopes, such as sialyl Lewis antigen (SLe) and sialyl-Tn (STn), associated with increased expression of sialyltransferases, are prominent features of various cancers [55] and associated with poor prognosis in patients with breast [60], colorectal [61] and stomach cancers [62]. Further, the increased expression of sialylated Lewis-type blood group antigens, such as SLe^a^ and SLe^x^ potentiate cancer cell migration by binding to endothelial selectins [12,63]. Overexpression of SLe^a^ and SLe^x^ are common features of several carcinomas (e.g., lung, colorectal, gastric and pancreas) and associated with increased metastatic capacity [64,65,66] and an overall poor patient survival [67,68].

**Deregulated Sugar Donor and Nucleotide Sugar Transporters:** The addition of terminal sugar residues (sialic acid, fucose or galactose) in the Golgi apparatus, during *N*-glycan biosynthesis, requires the availability of the appropriate sugar donors, which are transported from the cytoplasmic side to luminal side of Golgi membrane, using specific nucleotide sugar transporters. Nucleotide sugar transporters are often deregulated in cancer. For example, mRNA expression of UDP-galactose transporter was significantly increased for colorectal cancer tissues relative to non-tumor tissues. This increase in UDP-gal transporter further induced expression of Thomsen-Friedenreich (TF) antigen, SLe^a^ and SLe^x^, in transfected colorectal cancer cells resulting in an increased adhesion of CRC cells to vascular E-selectins [69]. In a CRC study, significant reduction in mRNA expression of sulfate transporter DTDST (Diastrophic Dysplasia Protein) was observed in colorectal cancer cells when compared to non-malignant cells, which was accompanied with an increased SLe^x^ and reduced sialyl 6-sulfo Lewis^x^ expression levels, coupled with an enhanced cell growth rate [70].

**Competition between Glycosyltransferases:** Competition between glycosylation enzymes for the same oligosaccharide substrate can also influence the structure of the resultant glycan, leading to structural heterogeneity and aberrant glycosylation in cancer. One such example is the competition between GNTs (responsible for the antenna-branching of *N*-glycans) and GNT3 (catalyzes the bisecting GlcNAc addition to *N*-glycans). The presence of bisecting GlcNAc inhibits the addition of the β1,6-branched chain [71,72], which in turn leads to elongated polylactosaminic chains and increased formation of the terminal carbohydrate antigens (e.g., SLe^x^). Another example includes the competition between ST3Gal1 and core 2 GlcNAcT-1 (C2GnT1), the enzymes responsible for the synthesis of sialyl-T antigen and core 2 branching, respectively. In human breast cancer cells, ST3Gal1 expression, by transfection, has predominantly generated core 1 structures, even in the presence of C2GnT1 expression [73]. Similarly, expression of C2GnT1 in SW480 colorectal cancer cell line has been shown to down-regulate T antigen expression [74].

**Altered Expression of Glycosidases:** Among glycosidases with most frequently altered expression in cancer, are sialidases, which catalyze the removal of sialic acid residues from the glycoconjugates. Four types of human sialidases have been identified; Neu1, lysosomal; Neu2, cytosolic; Neu3, cell membrane and Neu4, mitochondrial [75]. Down-regulation of Neu1 has been shown to promote the metastatic potential of cancer cells while its over-expression in murine melanoma cells could reverse malignancy [76]. Similarly, over-expression of Neu2 was shown to reduce the invasion of cancer cells and was linked to a concomitant reduction of the sialylated structures, including GM3 and SLe^x^ [77].

### Altered N-Glycosylation in Colorectal Cancer

Although altered protein *N*-glycosylation is well recognized as a hallmark event in carcinogenesis [78,79] it is not clear whether such changes are a cause and/or consequence of cancer [80,81]. Mapping the altered patterns of *N*-glycosylation may improve our understanding of the molecular mechanisms underlying perturbed glycosylation frequently observed in cancer. The biological significance of altered glycosylation for cancer detection is further highlighted by the fact that several cancer biomarkers currently used in the clinic are glycoproteins e.g., Her2/neu in breast cancer, CA-125 in ovarian cancer, prostate specific antigen (PSA) in prostate cancer, carcinoembryonic antigen (CEA) and cancer antigen 19-9 (CA-19-9) in CRC [10,82]. A number of other glycoproteins have also been proposed as potential CRC markers, using proteomics and genomics techniques, including EGFR, spectrins, carcinoembryonic antigen-related cell adhesion molecules (CEACAM), junction plakoglobin (JUP) and cadherin 17 (CDH17) [83,84,85,86]. With recent advances in analytical glycosciences [87,88,89] glycomics is gaining momentum as a tool in cancer research and holds considerable promise to identify candidate glycan markers for various cancer types, including CRC.

In recent years, numerous studies have sought to investigate the regulation of glycosylation in cancer and other diseases. Using a wide range of analytical techniques, including quantitative glycomics and glycoproteomics, lectin blotting, lectin glycoarray, immunohistochemistry (IHC) and reverse transcriptase-polymerase chain reaction (RT-PCR) of glyco-enzymes, these studies have demonstrated the aberrant glycosylation patterns in cancer and their association with tumor development, metastasis and invasion [12]. Examples of some altered *N*-glycans observed in CRC are summarized in Table 1. The ability to distinguish the differences in the glycosylation patterns of glycoproteins between cancer and control patients underscores glycobiology as a promising field for identification of potential cancer biomarkers.

**Table 1:** *N*-glycan alterations reported in different studies.

Aim of the Study	Finding	Altered *N-*glycan Structures		Reference
To elucidate differential expression of β1,6-branching in two variants of HCT116 CRC lines (HCT116a (more aggressive subline) and HCT116b).	Increased expression of β1,6-linked GlcNAc branching in HCT116a.	**↑**	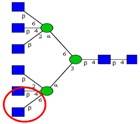	Li *et al.* [50]
To map the differences in *N*-glycans attached to lysosomal membrane glycoproteins isolated from CRC sublines exhibiting different metastatic potentials.	Increased poly-*N*-acetyl lactosamine (LacNAc) units and sialyl Le^x^, decreased fucosylation on LacNAc units of highly metastatic CRC cells relative to cells with less metastatic potential.	**↑**	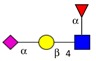	Saitoh *et al.* [90]
		**↑**	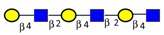	
		**↓**	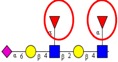	
To compare sialyltransferase activities in CRC tumor and adjacent normal mucosa.	Increased α2,6-sialyltransferase activity in CRC tumor relative to normal mucosa.	**↑**	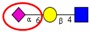	Dall’Olio *et al.* [55]
To compare the activity of sialyltranferases with different linkage specificities (α2,6- and α2,3-sialyltransferases) in different tissues including human CRC, normal mucosa, liver and liver metastases, and CRC patient serum samples.	Increased activity of α2,6-specific sialyltranferase in tumor tissue and serum of patients with metastatic tumors. α2,3-sialyltransferase activity was unchanged.	**↑**	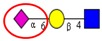	Gessner *et al.* [56]
To investigate the expression of α2,6- and α2,3-sialylation in CRC tumor tissues from different stages.	Increased α2,3-linked sialylation in stage I and II tumors, with a decrease in advanced CRC. Significant increase in α2,6-sialylation and in metastatic tumors.	**↑**	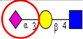	Vierbuchen *et al.* [91]
		**↑**	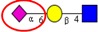	
To investigate the relationship between *N*-acetylglucosaminyl-transferase V (GnT-V) and metastasis in CRC tissues.	Expression of GnT-V significantly correlated with distant metastasis.	**↑**	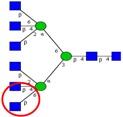	Murata *et al.* [51]
To compare the expression and activity of α1,6-fucosyltransferase in CRC tumor and healthy tissues.	Increased expression and activity of α1,6-fucosyltransferase expression and activity in CRC tumor compared to healthy tissues.	**↑**	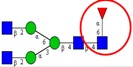	Muinelo-Romay *et al.* [92]
To compare the expression of sialo- and fucosyl-glycoconjugates in a panel of normal mucosa and adenocarcinoma samples, by lectin immunohistochemical analysis.	Increased expression of α2,6-linked sialic acid residues (as evident by strong staining of CRC tumor tissues with *Sambucusnigra* Lectin) in CRC tissue.	**↑**	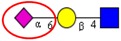	Fernández-Rodríguez *et al.* [93]
To detect glycosylation changes during colon epithelium differentiation and proliferation.	Significant decrease in high mannose type *N*-glycans and increase in atypical GlcNAc-ended *N*-glycans in differentiating HT-29 cells.	**↓**	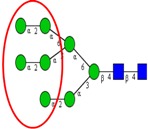	Vercoutter-Edouart *et al.* [94]
		**↑**	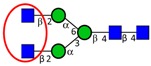	
To screen *N*-glycosylation changes in plasma samples from CRC patients.	Significantly higher levels of sialylation and fucosylation in patients with CRC or adenomas, compared to normal controls.	**↓**	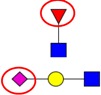	Qiu *et al.* [95]
To profile serum *N*-glycans in samples from healthy individuals and patients with CRC and adenomas, to identify potential *N*-glycan markers for prediction and detection of CRC.	Decreased total core α1,6 fucose residues and fucosyltransferase in CRC compared to adenomas and normal controls; Increased bi-galacto biantennary glycan and α1,3-fucosylated triantennary and decreased single and bi-galacto α1,6 fucosylated biantennary in CRC groups.	**↓**	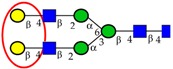	Zhao *et al.* [96]
		**↑**	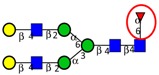	
		**↑**	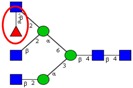	
To investigate alterations in sialylation and fucosylation in CRC patient tissues, by lectin immunohistochemical staining.	Predominant expression of α2,3 sialylated type 2 chain structures in CRC tissues associated with malignant transformation, in particular lymphatic spread.	**↑**	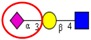	Fukasawa *et al.* [97]
Comparison of *N*-glycan profiles from a panel of CRC tumor tissues and corresponding control colon tissues, by hydrophilic interactionliquidchromatography and MALDI-TOF-MS.	Significant increase in sulfated, paucimannosidic and sialylated glycans, in particular *glycans*with*sialyl*Lewis type epitopes, and decrease in bisecting GlcNAc type *N*-glycans in CRC tumor tissues relative to normal tissues.	**↑**	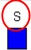	Balog *et al.* [35]
		**↑**	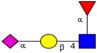	
		**↓**	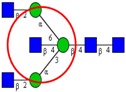	
Comparative *N*-glycan analysis of three pathologically and phenotypically different CRC cell lines (LIM1215, LIM1899, LIM2405).	Dominance of high mannose type and α2,6-sialylated glycans in all three cell lines; exclusive expression of bisecting GlcNAc and α2,3-sialylated *N*-glycans in metastatic (LIM1215) and aggressive (LIM2405) CRC cell lines, respectively.	**↑**	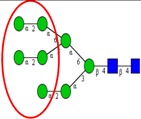	Sethi *et al.* [33]
		**↑**	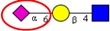	
Comparative glycomic analysis of CRC cell lines (SW1116, SW480, SW620, SW837, LS174) and CRC tissue samples.	Elevated high mannose type *N*-glycans in both CRC cell lines and tumor samples.	**↑**	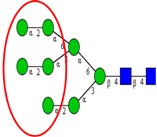	Chik *et al.* [98]
*N*-glycomic profiling of rectal adenomas and carcinomas by MALDI-TOF-MS, followed by IHC expression studies of sialyl Lewis a, and paucimannose glycans in a panel of CRC patients.	Mono-antennary, sialylated, paucimannose and small high mannose *N*-glycan structures were more common in carcinomas than in adenomas; correlation between poor prognosis and elevated expression of sialyl Lea and paucimannosidic *N*-glycans in CRC and advanced CRC, respectively.	**↑**	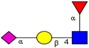	Kaprio *et al.* [99]
		**↑**	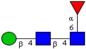	
		**↑**	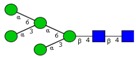	
Comparison of *N-*glycan profiles of membrane proteins from paired tumorigenic and adjacent non-tumorigenic CRC tissues.	Overrepresentation of high mannose, hybrid and paucimannosidic type *N*-glycans and under-representation of complex *N*-glycans in CRC tissues; higher sialylation, in particular α2,6-sialylation, in CRC tissues, coupled with down-regulation of α2,3-sialylation; high α2,3-sialylation and low bisecting β1,4-GlcNAcylation and Lewis-type fucosylation in mid-late stage CRC tissues, relative to early stage CRC;high bisecting β1,4-GlcNAcylation and low α2,3-sialylation in EGFR-positive tissues.	**↑**	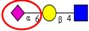	Sethi *et al.* [57]
		**↑**	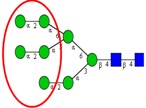	
		**↑**	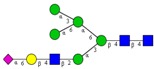	
		**↑**	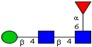	
		**↓**	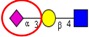	

## 5. LC-MS/MS-Based CRC *N-*Glycomics

### 5.1. Sample Handling for N-Glycan Analysis

**Figure 2:**
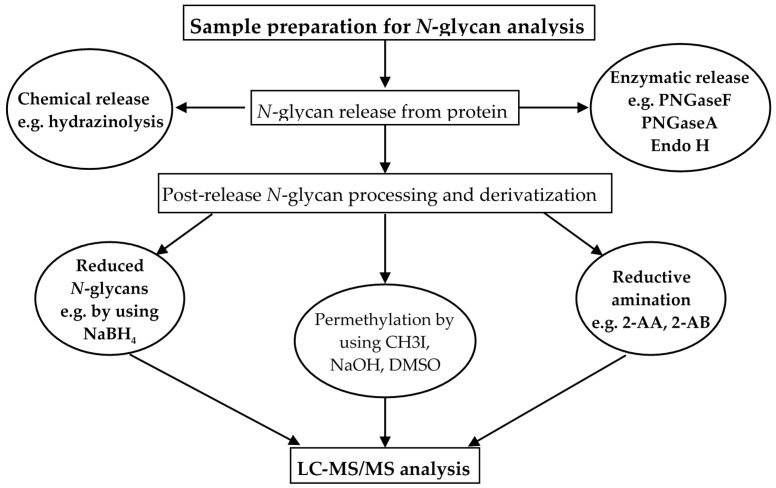
Sample preparation for *N*-glycan analysis by LC-MS/MS.

The two initial sample handling steps, typically integrated in glycomics, include *N*-glycan release from the protein carriers and post-release *N*-glycan derivatization prior to the LC-MS/MS analysis, as shown in Figure 2.

*N*-glycans can be released from glycoproteins using enzymatic or chemical methods. For the enzymatic release of *N*-glycans, several endoglycosidases and glycoamidases are routinely used. The most commonly used enzyme is peptide-*N*-glycosidase F (PNGase F), an asparagine deamidase that specifically hydrolyzes the bond between the reducing-end GlcNAc residue of the glycan moiety and the asparagine residue [100]. Although a rather non-specific enzyme, which can be used to cleave all *N*-glycans, PNGaseF cannot cleave core α1,3-fucosylated glycans; a glyco-feature commonly found in plants and insects [101]. Such glycan structures can be cleaved with PNGase A, which cleaves asparagine-linked *N*-glycans from glycopeptides containing 1,3-linked core fucose. Other endoglycosidases with variable degrees of specificity are also available, including the commonly used endoglycosidase H (Endo-H) that exclusively cleaves high mannose and hybrid type *N*-glycans (but not complex glycans) [100,102]. Also available are the cheaper and less specific chemical release methods such as hydrazinolysis [103,104], which cleaves the amide bond between the glycan and asparagine residue using hydrazine. While hydrazinolysis remains the preferred method for chemical release of glycans, it requires strict anhydrous and harsh conditions, including high temperature (95 °C) that leads to side reactions. In addition, it requires a reacetylation step in case of sialic acid and *N-*acetyl-amino sugars and specialized instrument to handle hydrazine [104,105].

Released *N*-glycans can be analyzed with or without derivatization in the free native form (aminated or hydroxylated in the reducing-end) or following a simple reduction step. The reduction of the glycan reducing-end is commonly carried out by sodium borohydride (NaBH_4_) treatment that locks the α- and β-anomers of the reducing-end to a single sugar alditol without isomer rearrangement. Although this simple method has a near-complete derivatization efficiency [104], and has been efficiently utilized for *N*-glycomic studies in different cancers, including CRC [33,57,98,106,107], a major drawback of this method compared to the derivatization methods is the absence of a chromophore for UV detection. As a result, native/reduced free glycans often have lower HPLC-UV/fluorescence and MS sensitivity than derivatized glycans e.g., obtained by permethylation [108,109] or reductive amination, using chromophores such as 2-aminobenzamide (2-AB) and 2-aminobenzoic acid (2-AA) [104,110].

Permethylation is one of the most widely used methods for glycan derivation that involves replacing the hydroxyl, amine and carboxyl groups with methyl groups, conferring hydrophobicity on glycan residues. Methylated glycans ionize more efficiently than their native counterparts, and due to their hydrophobic nature are easily separated from salts and other impurities that may affect the MS analysis. Moreover, permethylation enables simultaneous analysis of both acidic and neutral glycans in the positive ion mode, as the sialic acid residues on the acidic glycans are stabilized [108]. In an investigation of membrane *N*-glycoproteins from HT-29 colon carcinoma cells, *N*-glycans were first derivatized by permethylation, followed by MALDI-MS analysis [94]. This approach provided an overall compositional analysis of the *N*-glycans, but did not generate a detailed description of the glycoform structure heterogeneity. Tandem MS of permethylated glycans could be used as an alternative to obtain detailed structural and linkage information for glycans.

Labeling of the free reducing ends of glycans can also be achieved by reductive amination, which involves fluorescent derivatization of glycans. The most commonly used fluorescent labels include 2-aminobenzamide (2-AB), 2-aminobenzoic acid (2-AA), 2-aminopyridine (PA), 2-aminonaphthalene trisulfonic acid (ANTS), and 1-aminopyrene-3,6,8-trisulfonic acid (APTS). This method enables highly sensitive detection and high-resolution analysis of oligosaccharides [104,110], and coupled with LC separation is effectively used for quantitative *N-*glycomics. In a recent study, profiled *N*-glycosylation patterns of CRC and corresponding control tissues were profiled by labeling of released glycans with *2*-AB, followed by both hydrophilic interaction liquid chromatography (HILIC) and MALDI-TOF-MS analysis. Moreover, for structure elucidation, information from both positive mode ESI-ion trap-MS/MS and negative mode MALDI-TOF/TOF-MS were combined, providing a high-resolution structural determination of the *N*-glycans [35]. Similar fluorescent labeling strategies were also utilized for *N*-glycan profiling in other cancers such as, lung and breast cancer [111,112].

### 5.2. LC-Based Separation of N-Glycans

Recent advances in LC-MS glyco-analytical technologies have enabled detailed and accurate structural characterization of protein *N*-glycosylation. Although modern mass spectrometry has become a powerful tool for glycan detection, the extensive structural complexity and heterogeneity of the *N*-glycans requires separation of the glycans prior to MS detection, to achieve a more complete structural characterization. Established approaches used for off- or on-line separation of *N*-glycans, prior to MS, include liquid chromatography (LC) and capillary electrophoresis (CE). Due to its direct hyphenation to MS, LC is extensively used for glycomics analysis. Modern LC-based methods for glycomics include reverse phase (RP) [113,114,115,116], HILIC [115,117,118] and porous graphitized carbon (PGC) LC.

Reverse phase chromatography (RPC) has traditionally been used for analysis of glycans and glycoconjugates [116]. Oligosaccharides are separated on a C-18 column in order of hydrophobicity, with binding to the column decreasing with increasing size and polarity of the glycans. Native oligosaccharides are poorly retained on RPC due to their hydrophilic nature, requiring pre-derivatization by permethylation or fluorescent tagging to increase their hydrophobic character, to enhance separation [113]. Fluorescent labelling of *N*-glycans by 2-AB and 2-AA leads to efficient ionization and effective separation of structural isomers by RP-LC-MS/MS analysis [114,115].

HILIC is another widely applied LC technique for glycan separation. It is a variation of normal phase chromatography that separates native glycans based on hydrophilicity, polarity, size, charge and composition [115,117]. This method can be used with both native and derivatized glycans, and both neutral and acidic glycans can be separated with high reproducibility of retention times [119].

High pH anion exchange chromatography (HPAEC) coupled with pulsed electrochemical detection (PED) is another useful technique for carbohydrate determination, in which separation is based on the weakly acidic properties of sugar molecules [120]. The strong basic conditions partially deprotonates the hydroxyl groups on glycans, resulting in partial negative charges on glycans, which is used for separation of glycans, facilitated by interaction of oxyanions of oligosaccharides and amino groups of stationary phase column resin.

PGC-LC is a powerful separation technique, first introduced by Gilbert *et al*. in 1981 [121] and later modified by Knox *et al*. in 1986 [122]. It requires minimal sample preparation since no chemical derivatization of the glycan compounds is required. Coupling of PGC–LC with MS provides a powerful tool for detection and characterization of native and reduced glycans, with released glycans detected either as positively or negatively charged species depending on the type of solvent used for separation. A unique feature of this method is the high separation power for structural and linkage isomers, which in conjunction with MS, allows separate analysis of compounds exhibiting exactly the same *m/z* [123]. Certain *N-*glycan features influence the retention and elution behavior of PGC. For example, bisecting GlcNAc*-*containing *N*-glycans elute much earlier than *N*-glycans without this determinant. Similarly, α2,6-linked sialic acid residues elute earlier than isobaric *N*-glycans displaying α2,3-sialylation. These features make PGC–LC–ESI–MS/MS a very powerful tool for investigation of disease-specific glycan structures and features [123].

**Figure 3:**
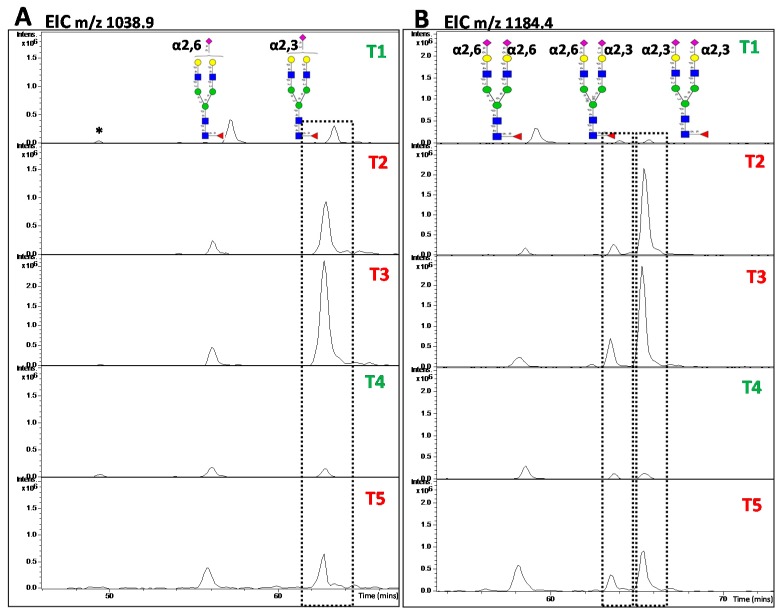
Extracted Ion Chromatograms (EIC) for mono- and di-sialylated biantennary complex type *N*-glycans (**A**) *m*/*z* 1038.9^2−^ and (**B**) *m*/*z* 1184.4^2−^ showing the separation power of PGC for α2,3 and α2,6-sialylation. Different ratios for α2,3 and α2,6-sialylation were observed between EGFR^+^ CRC (green, T1,T4) and EGFR^−^ CRC tissues (red, T2, T3 and T5) [57]. *, a low abundant glycan isomer.

The separation power of PGC has been successfully used in various cancer studies for separation of isomeric glycans. Sethi *et al.* [57] successfully applied this feature to demonstrate the differences in α2,6 and α2,3- linked sialic acid residues between EGFR^+^ and EGFR^−^ CRC tumor tissues, Figure 3. Similarly, several other studies utilized PGC-based retention time to determine the differences in the expression of α2,6- and α2,3-linked sialic acid residues in other cancers [33,106,107]. The high separation power of PGC-LC is not limited to sialylated residues and has also been extended to high mannose isomers and complex *N*-glycans structures such as Lewis^x/a/y/b^ structures [34,124]. The separation and detection of the individual topology/branching and linkage isomers is crucial in disease-centric research where alterations may occur only in certain determinants.

### 5.3. MS and MS/MS of N-Glycans

Ionization of biomolecules of interest into ions is a critical step in MS during which a neutral molecule (M) is converted into an ion by the addition or removal of electrons, acquiring positive or negative charges (e.g., M−H^−^ or M+H^+^). Some ionization techniques are very energetic, causing extensive fragmentation, while others are softer, producing spectra with less fragmentation, maintaining an intact molecule. Modern ionization methods for proteomics are electrospray ionization (ESI), first introduced by Dole *et al.* in 1968 [125] and later modified by Fenn *et al.* in 1989 [126], and matrix-assisted laser desorption ionization (MALDI), developed in the laboratories of Karas and Hillenkamp [127] and Tanaka *et al.* [128].

ESI is a liquid phase ionization technique, in which analytes in solution are sprayed as droplets directly into the mass spectrometer. ESI is particularly suited to the study of biomolecules such as peptides, glycans and proteins due to its ability to produce multiple charged species without extensive fragmentation. This allows larger molecules to be analyzed in the relatively low *m*/*z* range (*m*/*z* 500–1500) where most modern mass analyzers show optimal performance. The sensitivity of the ion detection is highly dependent on the analyte concentration, which is intrinsically linked to the solvent flow-rate with high sensitivity achieved by reducing the ESI flow rate to nanoliter-per-minute [129]. The ESI process is tolerant to low levels of salts and detergents, however these substances can form adducts that reduce analyte ion formation and signal suppression, resulting in ambiguous molecular mass determination [130].

MALDI, on the other hand, is a solid phase-based ionization technique, which produces ions by irradiating the solid sample mixture, dissolved in an organic matrix compound, with a pulsed laser beam, typically UV or IR. Similar to ESI, MALDI generates gas-phase ions, but unlike ESI-generated ions which carry multiple charges, MALDI-generated ions are only singly charged. MALDI can also tolerate low levels of salt, buffers and detergents, but data quality and sensitivity may be compromised [131]. Some limitations associated with using MALDI include low reproducibility and strong dependence on sample preparation methods [132,133]. MALDI and ESI-based approaches have been used for in-depth glycomic profiling of colorectal cancer, providing detailed *N*-glycan structural information [35,57,94].

Elucidation of *N*-glycan structures requires information on the accurate molecular mass and the fragmentation pattern (MS/MS or MS^n^) of the intact glycan of interest. MS/MS in particular has become an essential tool for structural glycomics, which provides structural information on the glycan of interest in only a single round of fragmentation. The main fragmentation mode of glycans is CID. ETD and ECD, which are useful for characterization of glycopeptides and proteins, are rarely used to analyze released glycans [134]. CID induces two types of cleavage in glycans; glycosidic cleavages which cleave bonds between two neighboring monosaccharide residues, and cross-ring cleavages that fragment two bonds within the same sugar residue. Glycosidic cleavages provide information on the monosaccharide composition and branching, while the latter usually provides more details on linkages and bonds [135]. CID fragmentation can be performed in either low or high energy, producing different fragmentation patterns. Low energy CID mainly generates glycosidic cleavages while the cross-ring cleavages are either absent or present in low abundance, which can limit the detailed structural assignment. High energy CID, on the other hand, may provide extensive fragmentation and yield informative, but possibly harder to interpret, *N-*glycan fragments [135].

**Figure 4:**
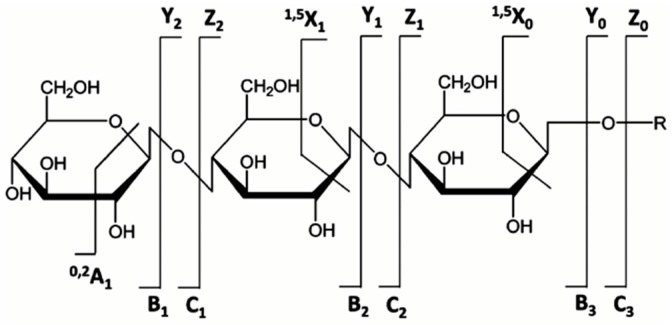
Nomenclature for the fragmentation of glycans; A and X ions represent cross-ring fragments and B, C, Y and Z are glycosidic fragment ions. (Adapted from Domon and Costello) [136].

Nomenclature of glycan fragments*—*The nomenclature for glycan fragmentation was first introduced by Domon and Costelloin [136], Figure 4, and still widely used to annotate the MS-generated glycan structures. Fragment ions of the intact reducing-end of the glycans are termed X, Y and Z, and the fragment ions containing the intact non-reducing-end of the glycans are termed A, B and C. A and X ions represent cross-ring fragments, while B, C, Y and Z ions are fragments arising from glycosidic bond cleavages. Subscripts indicate the number of individual sugar residues from the reducing-/non-reducing-end, whereas superscripts prior to the fragment letter indicate the cleavage position of the cross-ring fragment counted in a clockwise manner in the sugar ring.

**Figure 5:**
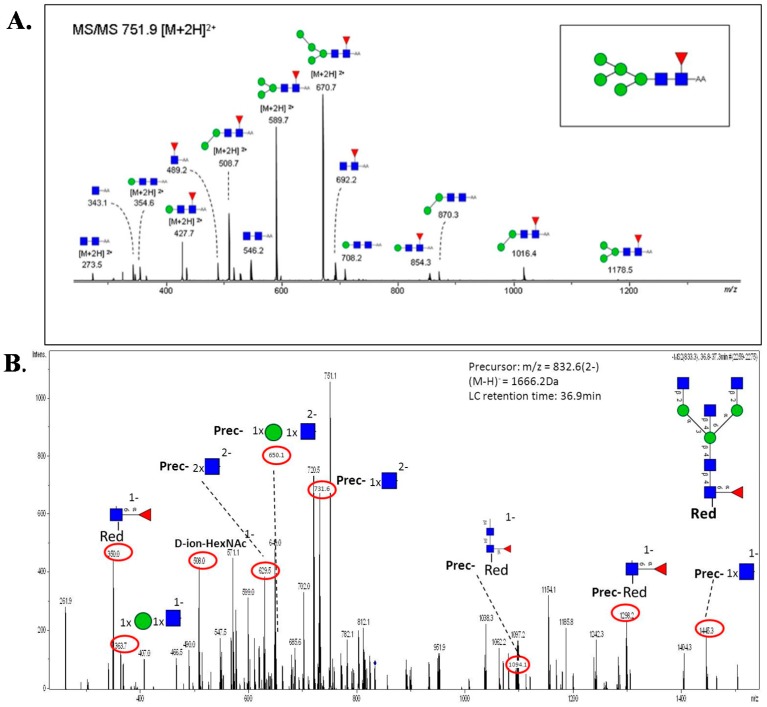
(**A**) Positive ion MS/MS fragmentation pattern of core fucosylated high mannose *N*-glycan type structure at *m*/*z* 751.9 [M + 2H]^2+^ [35]; (**B**) Negative ion MS/MS fragmentation pattern of core fucosylated bisecting *N*-glycan type structure at *m*/*z* 832.6 [33].

Glycan fragmentation patterns vary depending on the ion acquisition polarity mode. In positive mode, ionization of glycans results in the formation of [M+H]^+^ and [M+Na]^+^ pseudomolecular ions and adducts. CID fragmentation of glycans generates abundant B/Y type glycosidic fragments. Although the B/Y ions facilitate glycan sequencing, they provide little information on linkage and positional isomer information [137]. Derivatization, such as permethylation, coupled with tandem MS have been employed for elucidation of isomer structure [138,139]. Figure 5A shows the MS/MS fragmentation spectrum of an unusual core fucosylated high mannose structure (*m*/*z*= 751.9), generated in positive ion mode, observed in CRC tumor tissues [35]. The MS/MS fragment ions at *m*/*z*= 692.2 and 854.3 are diagnostic for the presence of core fucose, while *m*/*z*= 670.7, 589.7, 508.7 and 427.7 indicate subsequent loss of mannose residues.

Negative ion fragmentation of *N*-linked glycans produce abundant A-type cross-ring cleavages of the core GlcNAc residues, indicative of branch positions. Harvey [140] provided detailed fragmentation analysis of high mannose, hybrid and complex type *N*-glycans in negative mode. A characteristic feature of negative ion mode analysis is the generation of specific diagnostic fragment ions, useful for identification or to confirm certain glycan structural determinants. For example, the occurrence of a bisecting GlcNAc residue can be determined by the presence of an abundant [D-221]^−^ ion (e.g., *m*/*z*= 508 or 670) or detection of Z_1_ (*m*/*z*= 350) and Z_2_ (*m*/*z*= 553) ions as diagnostic ions for α1,6-core fucosylation [140]. Figure 5B shows a negative ion mode MS/MS fragmentation pattern of a unique bisecting type *N*-glycan structure (*m*/*z*= 832.6) observed exclusively in a metastatic CRC cell line [33]. Presence of D-221 at *m*/*z*= 508 and core fucose at *m*/*z*= 350 and subsequent loss of mannose and GlcNAc at *m*/*z*= 364 confirm the bisecting type and core fucosylated *N*-glycan structure.

## 6. Quantitative Glycomics

Quantitative determination of glycans can provide additional information for glycomics studies. Similar to quantitative proteomics, quantitative glycomics involves either a labeling approach or a label-free strategy. Both approaches provide relative quantitation of the glycan species within a sample (glycoprofile) and comparison of the resulting glycoprofiles between multiple glycomes. In the label-assisted approach, isotopic labels are incorporated metabolically or chemically into the glycans of interest prior to LC-MS/MS analysis [141]. Reductive amination with isobaric chromophores or permethylation with ^12^C/^13^C methyl iodide are widely used in labeling experiments [142]. In the widely used label-free approach, relative quantitation is commonly achieved using ion intensities obtained from extracted ion chromatograms of all the observed charge states of the identified glycans. From these intensity-based values, the relative abundance of the individual glycan is usually presented as a fraction (percentage) of the total glycome [35,57,99]. Glyco-profiling provided by the relative quantitation method can introduce potential inaccuracies/errors at several steps in the workflow e.g., sample preparation, derivatization and enrichment, matrix effects in complex samples or variabilities introduced at the LC-MS/MS step caused by irreproducible LC retention time or ionization bias towards certain glycoforms. In labelling approach, relative quantitation of the glycoform identified in different glycomes (samples) is determined by the ion ratios of the individual glycoforms. However, in most biological/disease-centric glycomics, this feature adds little value (except for shorter analysis time and possibly higher quantitative accuracy) relative to the conventional comparative glycoprofiling due to the inherent loss of quantitative information on the protein backbone [143].

## 7. Bioinformatics Tools and Glycome-Centric Databases and Resources

Despite extensive efforts dedicated to automate the annotation of LC-MS/MS-based glycan data, glycan spectral assignment still depends vastly on manual and expert data interpretation. Automatic methods for annotation of glycan data and assignment of glycan structures have been developed. One such tool is Cartoonist, which annotates *N*-glycans in MALDI-MS data by matching theoretical glycan masses to experimental masses [144]. However, it assigns glycan structures based on the molecular mass, which may potentially provide incorrect annotation. The recently developed GlycoWorkbench [145] is another bioinformatics tool for interpretation of MS data for glycans [146]. It supports multiple data formats from a variety of MS instrument platforms and has a glycan drawing tool (Glycan Builder) interface that allows users to define specific glycan structures and substructures to be annotated. This software is useful for the analyses of both glycan MS and MS/MS data. The online GlycoMod software [147] (available from the ExPASy website) is also a frequently used tool for elucidation of potential glycan monosaccharide composition based on MS-derived molecular masses [148]. Other glycan analysis tools used for MS and/or MS/MS data analysis include STAT, SysBioWare, Glycolyzer, SimGlycan and Glyco-Peakfinder, some of which are publicly available.

In addition to these (semi) automated tools for analysis of glycan data from LC-MS/MS data, efforts have been made towards integrating a number of glycomics-centric databases into a common platform called UnicarbKB [149] and UnicarbDB [150], which act as centralized data repositories for glycan data and work on the interface with the already available glycomics (GlycomeDB, EUROcarbDB) and proteomics (Uniprot) databases [151,152]. Other glyco-related databases include Glycosciences.de, Kyoto Encyclopaedia of Gene and Genomes Glycans (KEGG Glycans), and the consortium for functional glycomics (CFG) and the more recently developed GIyTouCan (http://glytoucan.org/) [153].

## 8. Conclusions

This review provides an overview of the capacity of present-day LC-MS/MS-based *N*-glycomics, and the associated challenges, for accurate mapping of the cancer glycome (including CRC), to gain unique and novel insights into the cancer-associated glycan alterations.

Glycans have great potential as biomarkers for diseases including cancer because of their association with important carcinogenic processes, including tumor progression and metastasis. The various alterations in *N*-glycan patterns, reported in different cancers, highlight the importance of *N-*glycome as a molecular signature in cancer. Deciphering the “glyco-code”, using advanced and highly sensitive LC-MS/MS-based glycomics approach and platform may provide valuable information, to allow a better understanding of the biomolecular deregulations associated with altered *N*-glycosylation in cancer.

In recent years, glycomics has revolutionized the field of biomarker discovery by providing considerable insights into disease mechanisms and molecular regulation of protein glycosylation associated with specific diseases [154]. Coupled with advances in mass spectrometry and bioinformatics, glycomics identified multiple potential glycan-based biomarkers in various cancers, including, CRC [99,111,112,155]. However, significant challenges are still associated with transition for proposed glycan biomarkers from discovery to the clinical phase, including: (i) extensive heterogeneity of the detected glycoforms in the often highly complex sample matrix; (ii) sensitivity of the glycosylation machinery to the biochemical environment, including the impact of acute phase reactions and inflammation. Certain unusual paucimannosidic type *N*-glycans, which are characteristic features of human neutrophils, linked to inflammation and important elements of tumor microenvironment, were observed in CRC tumor glycome [35,37,57,156].

## Abbreviations

The abbreviations used are: CRC, colorectal cancer; CID, collision-induced *dissociation*; *ESI*, electrospray ionization; GlcNAc, *N*-acetylglucosamine; GnT-III, *N*-acetylglucosaminyl transferase III; IHC, LC, liquid chromatography; MS, mass spectrometry; MS/MS, tandem mass spectrometry; PGC, porous graphitized carbon; PNGase F, peptide*-N*-glycosidase F; HILIC, Hydrophilic interaction liquid chromatography; RPC, Reverse phase chromatography.
